# Supplementary material for: Widespread erosion on high plateaus during recent glaciations in Scandinavia
Source: Nat Commun. 2018 Feb 26;9:830. doi: 10.1038/s41467-018-03280-2 (PMC5827020; doi:10.1038/s41467-018-03280-2)
Supplement: Supplementary file 1 — Supplementary information [file 41467_2018_3280_MOESM1_ESM.pdf]

# Supplementary Figures and Tables

## Widespread erosion on Scandinavian plateaus during recent glaciations

Andersen et al.

This file contains the following supplementary figures and tables:


- Supplementary Figure 1 : Published cosmogenic nuclide data from Southern Norway
- Supplementary Figure 2 : Map overview of apparent $^{10}Be$ exposure ages and $^{26}Al/^{10}Be$ ratios, Sognefjord.
- Supplementary Figure 3 : Sensitivity tests of inverse-modelled erosion rates
- Supplementary Figure 4 : Field photographs of selected samplesites

- Supplementary Table 1 : Sample details and apparent $^{10}Be$ and $^{26}Al$ ages
- Supplementary Table 2 : Laboratory and AMS data
- Supplementary Table 3 : Inverse-modelled erosion rates

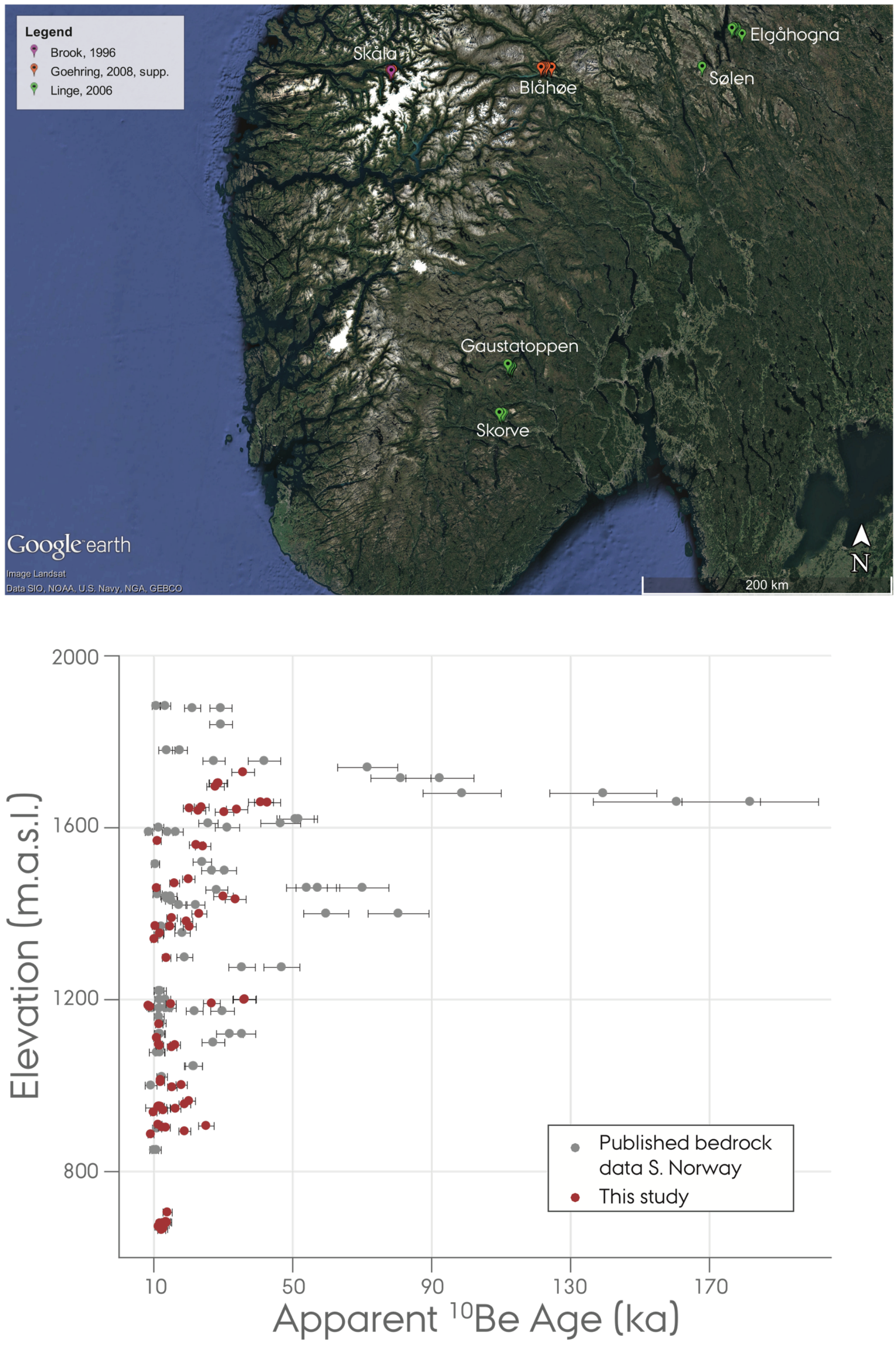


**Supplementary Figure 1:** Top: Google Earth image of southern Norway showing sample localities of previously published bedrock data[1-3]. Bottom: $^{10}Be$ apparent exposure ages from this study as well as previously published bedrock data from southern Norway. All apparent ages are re-calculated using the scaling scheme from Borchers et al. 2016 (Sup. Ref. 4), errorbars show external uncertainty from the online calculators formerly known as the CRONUS Earth online calculators[5].

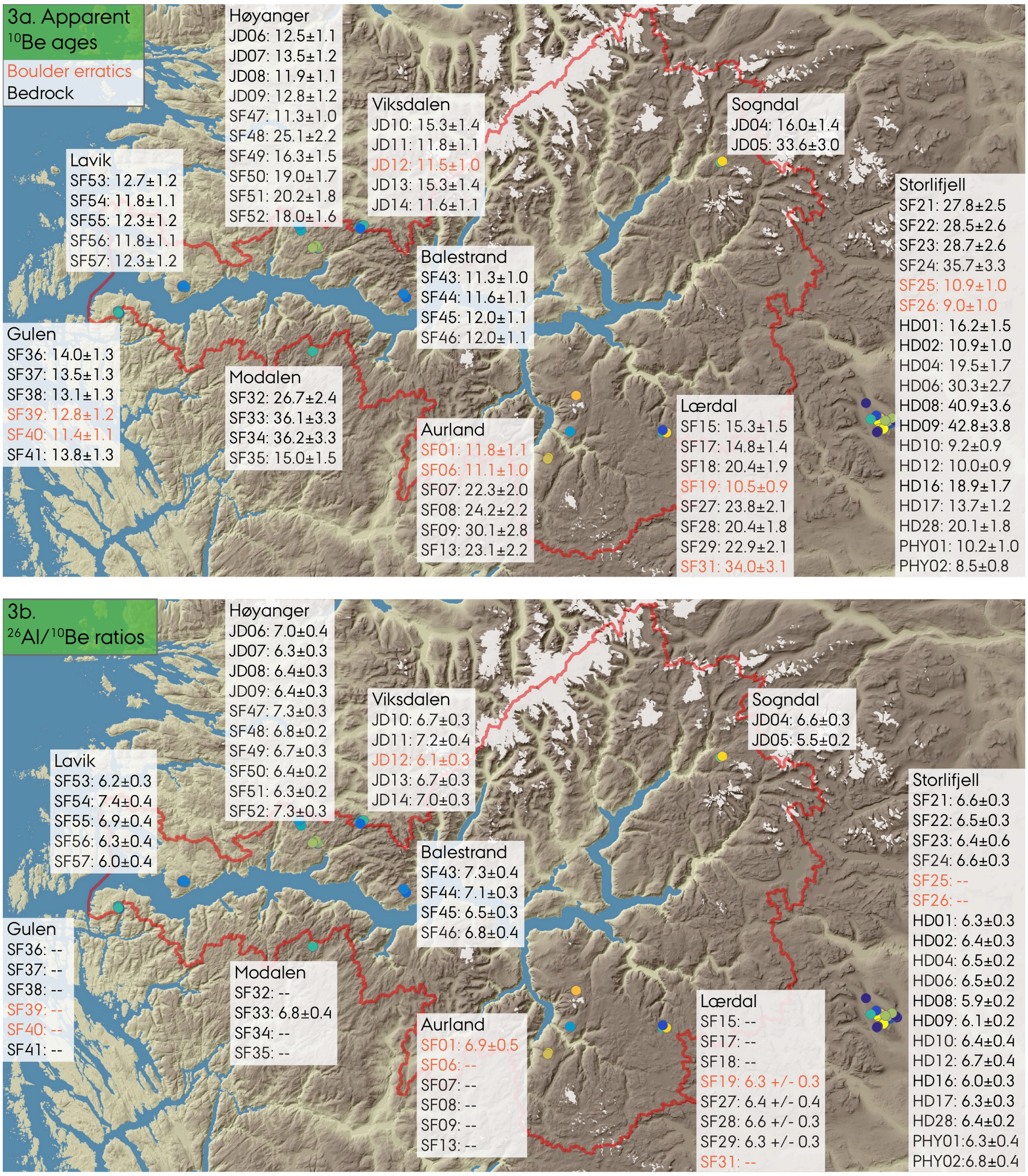


**Supplementary Figure 2: (a)** Map overview of $^{10}$Be apparent ages (kyr) of all bedrock (n=60) and boulder erratic (n=9) samples with external uncertainties. Sognefjord catchment delineated in red, **(b)** $^{26}$Al/$^{10}$Be ratios from samples were both nuclides were measured. Map created with ESRI ArcGIS software from a digital elevation model freely available at www.geonorge.no.

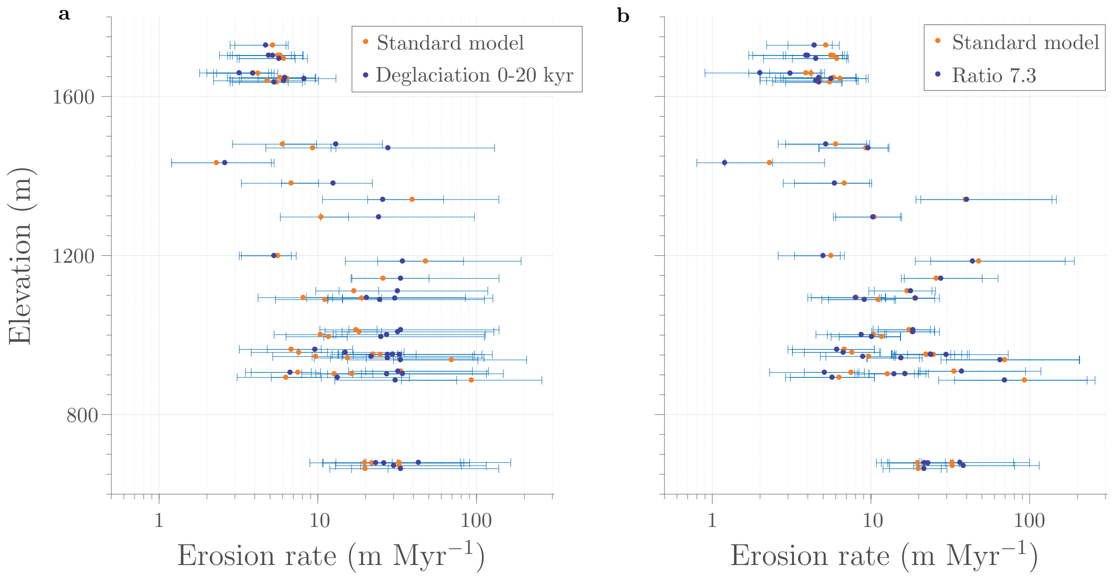


**Supplementary Figure 3:** Sensitivity tests of Markov chain Monte Carlo inverse-modelled erosion rates (m Myr$^{-1}$) towards **(a)** choice of last deglaciation parameters and **(b)** surface production ratio. The 'standard' model has site-specific deglaciation ages retrieved from Stroeven et al. 2015 (+- 0.5 kyr) and surface production ratio of 6.75 (see Methods). Errorbars represent the interquartile range in total erosion rates of all accepted models.

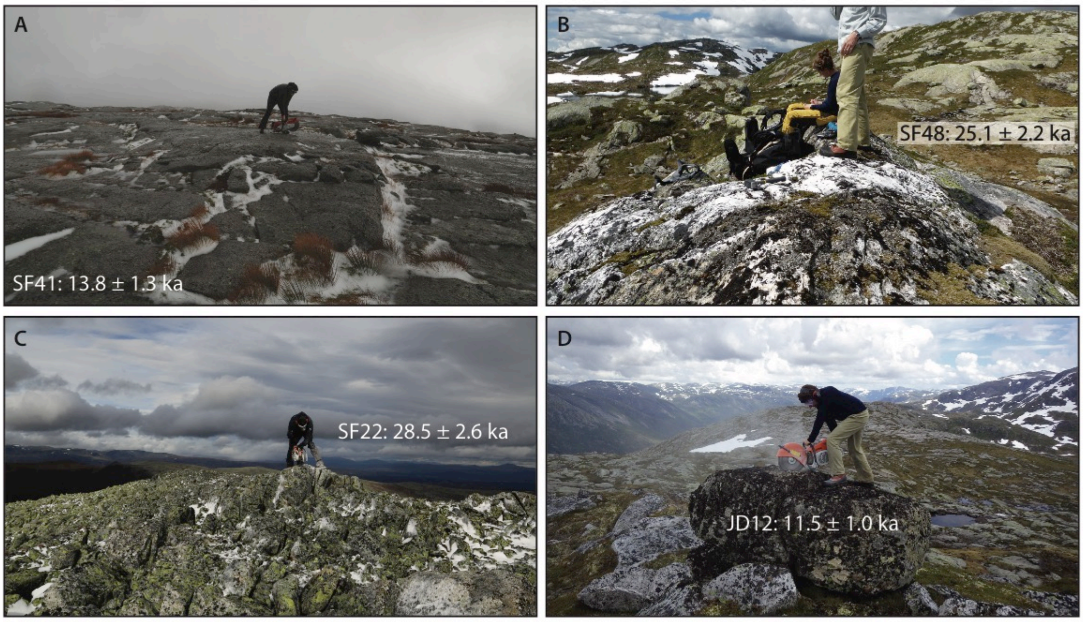


**Supplementary Figure 4:** Photographs of samples collected in summer 2012 and 2014, illustrating surface morphologies sampled in the field area: **(a)** and **(b)** glacially sculpted bedrock sites with variable weathering degree, **(c)** bedrock outcrop in blockfield, and **(d)** boulder erratic. Ages are given in ka with external uncertainty from the online calculators formerly known as the CRONUS Earth online calculators[5].

## Supplementary table 1. Apparent ages and sample data

| Sample ID | Type | Elevation (m) | Latitude* | Longitude* | Thickness (cm) | Shielding correction | Processing lab** | AMS analysis** | 26Al Age (kyr)*** | Unc. | 10Be Age (kyr)*** | Unc. | 26Al/10Be ratio | Unc. |
|---|---|---|---|---|---|---|---|---|---|---|---|---|---|---|
| HD01 | Bedrock | 1094 | 60,9954 | 8,7263 | 3,2 | 0,99 | Ber/Aar | AARAMS | 15,2 | 1,4 | 16,2 | 1,5 | 6,3 | 0,3 |
| HD02 | Bedrock | 1111 | 60,9964 | 8,7246 | 4,0 | 0,99 | Ber/Aar | AARAMS | 10,4 | 1,0 | 10,9 | 1,0 | 6,4 | 0,3 |
| HD04 | Bedrock | 1382 | 61,0020 | 8,7134 | 3,0 | 1,00 | Ber/Aar | AARAMS | 18,8 | 1,7 | 19,5 | 1,7 | 6,5 | 0,2 |
| HD06 | Bedrock | 1636 | 60,9839 | 8,6788 | 4,1 | 1,00 | Ber/Aar | AARAMS | 29,4 | 2,6 | 30,3 | 2,7 | 6,5 | 0,2 |
| HD08 | Bedrock | 1659 | 60,9836 | 8,6969 | 3,8 | 1,00 | Ber/Aar | AARAMS | 35,9 | 3,2 | 40,9 | 3,6 | 5,9 | 0,2 |
| HD09 | Bedrock | 1658 | 60,9845 | 8,6985 | 3,9 | 1,00 | Ber/Aar | AARAMS | 38,7 | 3,4 | 42,8 | 3,8 | 6,1 | 0,2 |
| HD10 | Bedrock | 886 | 61,0016 | 8,7555 | 2,3 | 1,00 | Ber/Aar | AARAMS | 8,6 | 0,8 | 9,2 | 0,9 | 6,4 | 0,4 |
| HD12 | Bedrock | 938 | 61,0044 | 8,7499 | 1,0 | 1,00 | Ber/Aar | AARAMS | 9,9 | 0,9 | 10,0 | 0,9 | 6,7 | 0,4 |
| HD16 | Bedrock | 893 | 61,0100 | 8,7310 | 3,3 | 0,99 | Ber/Aar | AARAMS | 16,7 | 1,5 | 18,9 | 1,7 | 6,0 | 0,3 |
| HD17 | Bedrock | 1297 | 61,0033 | 8,6357 | 1,8 | 1,00 | Ber/Aar | AARAMS | 12,7 | 1,2 | 13,7 | 1,2 | 6,3 | 0,3 |
| HD28 | Bedrock | 1480 | 61,0026 | 8,7024 | 0,8 | 0,99 | Aar | AARAMS | 18,9 | 1,7 | 20,1 | 1,8 | 6,4 | 0,2 |
| JD04 | Bedrock | 1470 | 61,5367 | 7,8215 | 2 | 1,00 | Aar | AARAMS | 15,7 | 1,4 | 16,0 | 1,4 | 6,6 | 0,3 |
| JD05 | Bedrock | 1433 | 61,5397 | 7,8302 | 2 | 1,00 | Aar | AARAMS | 27,4 | 2,5 | 33,6 | 3,0 | 5,5 | 0,2 |
| JD06 | Bedrock | 903 | 61,2704 | 5,9341 | 3,8 | 1,00 | Aar | AARAMS | 12,9 | 1,2 | 12,5 | 1,1 | 7,0 | 0,4 |
| JD07 | Bedrock | 902 | 61,2707 | 5,9338 | 3 | 1,00 | Aar | AARAMS | 12,6 | 1,2 | 13,5 | 1,2 | 6,3 | 0,3 |
| JD08 | Bedrock | 951 | 61,2827 | 5,9246 | 3,8 | 1,00 | Aar | AARAMS | 11,3 | 1,0 | 11,9 | 1,1 | 6,4 | 0,3 |
| JD09 | Bedrock | 942 | 61,2816 | 5,9190 | 5 | 1,00 | Aar | AARAMS | 12,0 | 1,1 | 12,8 | 1,2 | 6,4 | 0,3 |
| JD10 | Bedrock | 996 | 61,2960 | 6,1975 | 2 | 1,00 | Aar | AARAMS | 15,1 | 1,4 | 15,3 | 1,4 | 6,7 | 0,3 |
| JD11 | Bedrock | 1093 | 61,2999 | 6,2060 | 4,5 | 1,00 | Aar | AARAMS | 12,5 | 1,2 | 11,8 | 1,1 | 7,2 | 0,4 |
| JD12 | Boulder | 1095 | 61,2997 | 6,2061 | 5,5 | 1,00 | Aar | AARAMS | 10,3 | 1,0 | 11,5 | 1,0 | 6,1 | 0,3 |
| JD13 | Bedrock | 1089 | 61,3011 | 6,2150 | 6 | 1,00 | Aar | AARAMS | 15,2 | 1,4 | 15,3 | 1,4 | 6,7 | 0,3 |
| JD14 | Bedrock | 1142 | 61,2926 | 6,2022 | 3 | 1,00 | Aar | AARAMS | 11,9 | 1,1 | 11,6 | 1,1 | 7,0 | 0,3 |
| Phy01 | Bedrock | 1341 | 60,9782 | 8,6726 | 1,5 | 0,98 | Ber/Aar | AARAMS | 9,5 | 0,9 | 10,2 | 1,0 | 6,3 | 0,4 |
| Phy02 | Bedrock | 1186 | 60,9756 | 8,6701 | 2,3 | 0,98 | Ber/Aar | AARAMS | 8,5 | 0,8 | 8,5 | 0,8 | 6,8 | 0,4 |
| SF01 | Boulder | 1354 | 60,9042 | 7,2764 | 5,9 | 1,00 | Aar | AARAMS | 12,1 | 1,2 | 11,8 | 1,1 | 6,9 | 0,5 |
| SF06 | Boulder | 1569 | 60,9851 | 7,2817 | 3,6 | 1,00 | SUERC | SUERC | -- | -- | 11,1 | 1,0 | -- | -- |
| SF07 | Bedrock | 1560 | 60,9854 | 7,2829 | 2,6 | 0,99 | SUERC | SUERC | -- | -- | 22,3 | 2,0 | -- | -- |
| SF08 | Bedrock | 1556 | 60,9854 | 7,2834 | 2,6 | 1,00 | SUERC | SUERC | -- | -- | 24,2 | 2,2 | -- | -- |
| SF09 | Bedrock | 1440 | 60,8404 | 7,1919 | 3,9 | 1,00 | SUERC | SUERC | -- | -- | 30,1 | 2,8 | -- | -- |
| SF13 | Bedrock | 1399 | 60,8368 | 7,1893 | 3,0 | 1,00 | SUERC | SUERC | -- | -- | 23,1 | 2,2 | -- | -- |
| SF15 | Bedrock | 1390 | 60,9248 | 7,6945 | 3,1 | 1,00 | SUERC | SUERC | -- | -- | 15,3 | 1,5 | -- | -- |
| SF17 | Bedrock | 1370 | 60,9314 | 7,6945 | 4,1 | 1,00 | SUERC | SUERC | -- | -- | 14,8 | 1,4 | -- | -- |
| SF18 | Bedrock | 1369 | 60,9315 | 7,6945 | 4,4 | 1,00 | SUERC | SUERC | -- | -- | 20,4 | 1,9 | -- | -- |
| SF19 | Boulder | 1371 | 60,9311 | 7,6955 | 4,2 | 0,99 | Aar | AARAMS | 9,8 | 0,9 | 10,5 | 0,9 | 6,3 | 0,3 |
| SF21 | Bedrock | 1695 | 61,0072 | 8,6772 | 2,7 | 1,00 | SUERC | SUERC | 27,3 | 2,5 | 27,8 | 2,5 | 6,6 | 0,3 |
| SF22 | Bedrock | 1703 | 61,0080 | 8,6809 | 4,5 | 1,00 | SUERC | SUERC | 27,5 | 2,5 | 28,5 | 2,6 | 6,5 | 0,3 |
| SF23 | Bedrock | 1703 | 61,0062 | 8,6817 | 4,7 | 1,00 | SUERC | SUERC | 27,2 | 3,4 | 28,7 | 2,6 | 6,4 | 0,6 |

| Sample ID | Type | Elevation (m) | Latitude* | Longitude* | Thickness (cm) | Shielding correction | Processing lab** | AMS analysis** | 26Al Age (kyr)*** | Unc. | 10Be Age (kyr)*** | Unc. | 26Al/10Be ratio | Unc. |
|---|---|---|---|---|---|---|---|---|---|---|---|---|---|---|
| SF24 | Bedrock | 1729 | 61,0004 | 8,6691 | 4,3 | 1,00 | SUERC | SUERC | 34,9 | 3,2 | 35,7 | 3,3 | 6,6 | 0,3 |
| SF25 | Boulder | 1459 | 61,0122 | 8,6589 | 3,3 | 1,00 | SUERC | SUERC | -- | -- | 10,9 | 1,0 | -- | -- |
| SF26 | Boulder | 1183 | 61,0375 | 8,6067 | 3,3 | 1,00 | SUERC | SUERC | -- | -- | 9,0 | 1,0 | -- | -- |
| SF27 | Bedrock | 1647 | 60,9259 | 7,7125 | 4,7 | 1,00 | SUERC | SUERC | 22,6 | 2,3 | 23,8 | 2,1 | 6,4 | 0,4 |
| SF28 | Bedrock | 1645 | 60,9265 | 7,7098 | 4,5 | 1,00 | SUERC | SUERC | 19,8 | 1,9 | 20,4 | 1,8 | 6,6 | 0,3 |
| SF29 | Bedrock | 1639 | 60,9271 | 7,7085 | 4,9 | 1,00 | SUERC | SUERC | 21,3 | 2,0 | 22,9 | 2,1 | 6,3 | 0,3 |
| SF31 | Boulder | 1642 | 60,9258 | 7,7127 | 3,8 | 1,00 | SUERC | SUERC | -- | -- | 34,0 | 3,1 | -- | -- |
| SF32 | Bedrock | 1191 | 61,0074 | 6,0637 | 2,8 | 1,00 | SUERC | SUERC | -- | -- | 26,7 | 2,4 | -- | -- |
| SF33 | Bedrock | 1199 | 61,0082 | 6,0604 | 3,8 | 1,00 | SUERC | SUERC | 36,7 | 3,7 | 36,1 | 3,3 | 6,8 | 0,4 |
| SF34 | Bedrock | 1201 | 61,0087 | 6,0607 | 4,6 | 1,00 | SUERC | SUERC | -- | -- | 36,2 | 3,3 | -- | -- |
| SF35 | Bedrock | 1189 | 61,0083 | 6,0621 | 2,9 | 1,00 | SUERC | SUERC | -- | -- | 15,0 | 1,5 | -- | -- |
| SF36 | Bedrock | 705 | 61,0349 | 5,1555 | 4,6 | 1,00 | SUERC | SUERC | -- | -- | 14,0 | 1,3 | -- | -- |
| SF37 | Bedrock | 683 | 61,0343 | 5,1584 | 5,2 | 1,00 | SUERC | SUERC | -- | -- | 13,5 | 1,3 | -- | -- |
| SF38 | Bedrock | 675 | 61,0308 | 5,1631 | 5,5 | 1,00 | SUERC | SUERC | -- | -- | 13,1 | 1,3 | -- | -- |
| SF39 | Boulder | 666 | 61,0304 | 5,1639 | 4,2 | 1,00 | SUERC | SUERC | -- | -- | 12,8 | 1,2 | -- | -- |
| SF40 | Boulder | 672 | 61,0304 | 5,1628 | 5,4 | 1,00 | SUERC | SUERC | -- | -- | 11,4 | 1,1 | -- | -- |
| SF41 | Bedrock | 681 | 61,0311 | 5,1574 | 4,2 | 1,00 | SUERC | SUERC | -- | -- | 13,8 | 1,3 | -- | -- |
| SF43 | Bedrock | 951 | 61,1547 | 6,4532 | 3 | 1,00 | Aar | AARAMS | 12,2 | 1,1 | 11,3 | 1,0 | 7,3 | 0,4 |
| SF44 | Bedrock | 952 | 61,1544 | 6,4534 | 3 | 1,00 | Aar | AARAMS | 12,2 | 1,1 | 11,6 | 1,1 | 7,1 | 0,3 |
| SF45 | Bedrock | 1008 | 61,1615 | 6,4390 | 3,2 | 1,00 | Aar | AARAMS | 11,6 | 1,1 | 12,0 | 1,1 | 6,5 | 0,3 |
| SF46 | Bedrock | 1013 | 61,1615 | 6,4417 | 3,5 | 1,00 | Aar | AARAMS | 12,0 | 1,2 | 12,0 | 1,1 | 6,8 | 0,4 |
| SF47 | Bedrock | 909 | 61,2360 | 6,0212 | 2,5 | 1,00 | Aar | AARAMS | 12,2 | 1,1 | 11,3 | 1,0 | 7,3 | 0,3 |
| SF48 | Bedrock | 906 | 61,2375 | 6,0233 | 3 | 1,00 | Aar | AARAMS | 25,3 | 2,3 | 25,1 | 2,2 | 6,8 | 0,2 |
| SF49 | Bedrock | 946 | 61,2391 | 6,0192 | 2 | 1,00 | Aar | AARAMS | 16,1 | 1,5 | 16,3 | 1,5 | 6,7 | 0,3 |
| SF50 | Bedrock | 956 | 61,2426 | 6,0105 | 4 | 1,00 | Aar | AARAMS | 17,9 | 1,6 | 19,0 | 1,7 | 6,4 | 0,2 |
| SF51 | Bedrock | 964 | 61,2382 | 5,9966 | 3 | 1,00 | Aar | AARAMS | 18,7 | 1,7 | 20,2 | 1,8 | 6,3 | 0,2 |
| SF52 | Bedrock | 1001 | 61,2357 | 5,9965 | 2 | 1,00 | Aar | AARAMS | 19,5 | 1,8 | 18,0 | 1,6 | 7,3 | 0,3 |
| SF53 | Bedrock | 678 | 61,1093 | 5,4439 | 1,5 | 1,00 | Aar | AARAMS | 11,6 | 1,1 | 12,7 | 1,2 | 6,2 | 0,3 |
| SF54 | Bedrock | 679 | 61,1096 | 5,4414 | 1,5 | 1,00 | Aar | AARAMS | 12,9 | 1,2 | 11,8 | 1,1 | 7,4 | 0,4 |
| SF55 | Bedrock | 664 | 61,1094 | 5,4298 | 3,5 | 1,00 | Aar | AARAMS | 12,5 | 1,2 | 12,3 | 1,2 | 6,9 | 0,4 |
| SF56 | Bedrock | 672 | 61,1114 | 5,4307 | 3,5 | 1,00 | Aar | AARAMS | 10,9 | 1,1 | 11,8 | 1,1 | 6,3 | 0,4 |
| SF57 | Bedrock | 679 | 61,1130 | 5,4348 | 3,8 | 1,00 | Aar | AARAMS | 10,9 | 1,1 | 12,3 | 1,2 | 6,0 | 0,4 |

* Determined by handheld GPS, reference datum WGS84

** Ber = Bergen University, Aar = Aarhus University, SUERC=Scottish Universities Environmental Research Center, AARAMS=Aarhus AMS Centre

*** Age calculation of samples using the CRONUS online calculator (Balco et al. 2008). $^{10}$Be ages calculated with high-latitude sea-level spallation production rate of 4.01 at $g^{-1}$ $yr^{-1}$ (Borcher et al. 2016), $^{26}$Al ages calculated assuming surface production ratio of 6.75. All ages calculated with standard amosphere, zero erosion, a rock density of 2650 kg $m^{-3}$, and the time-independent Lal/Stone (2000) spallation scaling scheme and are normalized to the “07KNSTD” and “KNSTD” isotope ratio standardizations.

## Supplementary table 2. Laboratory and AMS data

| Sample ID | Quartz Mass (g) | Total Al (ug)* | Unc. | Laboratory blank Total Al (ug)* | Unc. | 10Be/9Be Ratio | Unc. | Laboratory blank 10Be/9Be Ratio | Unc. | 26Al/27Al Ratio | Unc. | Laboratory blank 26Al/27Al Ratio | Unc. | 10Be Conc. (atoms g^-1) | Unc. | 26Al Conc. (atoms g^-1) | Unc. |
|---|---|---|---|---|---|---|---|---|---|---|---|---|---|---|---|---|---|
| HD01 | 20,8093 | 520,4 | 6,8 | 991,2 | 1,7 | 2,44E-13 | 7,08E-15 | 1,63E-15 | 1,01E-15 | 2,11E-12 | 6,87E-14 | 5,75E-15 | 2,72E-15 | 185407 | 5521 | 1173583 | 41439 |
| HD02 | 21,7774 | 976,8 | 1,9 | 991,2 | 1,7 | 1,74E-13 | 5,74E-15 | 1,63E-15 | 1,01E-15 | 8,15E-13 | 3,23E-14 | 5,75E-15 | 2,72E-15 | 126073 | 4286 | 809930 | 32453 |
| HD04 | 22,0808 | 989,6 | 2,3 | 991,2 | 1,7 | 3,97E-13 | 9,73E-15 | 1,63E-15 | 1,01E-15 | 1,87E-12 | 5,05E-14 | 5,75E-15 | 2,72E-15 | 285721 | 7173 | 1860314 | 50752 |
| HD06 | 22,9704 | 1001,0 | 1,5 | 991,2 | 1,7 | 7,84E-13 | 1,35E-14 | 1,63E-15 | 1,01E-15 | 3,61E-12 | 7,19E-14 | 5,75E-15 | 2,72E-15 | 538145 | 9599 | 3505378 | 70214 |
| HD08 | 23,2185 | 921,2 | 2,4 | 991,2 | 1,7 | 1,08E-12 | 1,79E-14 | 1,63E-15 | 1,01E-15 | 4,92E-12 | 9,18E-14 | 5,75E-15 | 2,72E-15 | 737382 | 12607 | 4350417 | 82169 |
| HD09 | 24,8586 | 1345,7 | 1,1 | 991,2 | 1,7 | 1,20E-12 | 1,86E-14 | 1,63E-15 | 1,01E-15 | 3,88E-12 | 7,09E-14 | 5,75E-15 | 2,72E-15 | 770144 | 12328 | 4680093 | 85728 |
| HD10 | 21,9057 | 1114,3 | 2,3 | 991,2 | 1,7 | 1,26E-13 | 4,57E-15 | 1,63E-15 | 1,01E-15 | 5,07E-13 | 2,25E-14 | 5,75E-15 | 2,72E-15 | 89794 | 3415 | 570258 | 25736 |
| HD12 | 22,3500 | 956,4 | 1,8 | 991,2 | 1,7 | 1,47E-13 | 5,28E-15 | 1,63E-15 | 1,01E-15 | 7,25E-13 | 2,89E-14 | 5,75E-15 | 2,72E-15 | 103049 | 3838 | 686723 | 27792 |
| HD16 | 22,3136 | 910,4 | 2,0 | 991,2 | 1,7 | 2,57E-13 | 7,10E-15 | 1,63E-15 | 1,01E-15 | 1,20E-12 | 3,64E-14 | 5,75E-15 | 2,72E-15 | 182224 | 5170 | 1086794 | 33319 |
| HD17 | 23,4385 | 955,0 | 2,5 | 991,2 | 1,7 | 2,81E-13 | 7,14E-15 | 1,63E-15 | 1,01E-15 | 1,31E-12 | 4,20E-14 | 5,75E-15 | 2,72E-15 | 189168 | 4952 | 1182473 | 38437 |
| HD28 | 25,5717 | 1197,0 | 14,0 | 1043,7 | 9,9 | 4,84E-13 | 1,01E-14 | 3,92E-15 | 9,24E-16 | 1,96E-12 | 4,49E-14 | 1,88E-15 | 1,42E-15 | 321063 | 7161 | 2041108 | 52679 |
| JD04 | 26,1157 | 1338,3 | 13,7 | 993,8 | 8,4 | 3,99E-13 | 9,03E-15 | 3,33E-15 | 8,81E-16 | 1,48E-12 | 4,68E-14 | 2,13E-15 | 1,85E-15 | 255384 | 6139 | 1686823 | 56265 |
| JD05 | 14,7121 | 1538,5 | 12,8 | 993,8 | 8,4 | 4,53E-13 | 1,03E-14 | 3,33E-15 | 8,81E-16 | 1,22E-12 | 3,92E-14 | 2,13E-15 | 1,85E-15 | 517606 | 12493 | 2846109 | 94487 |
| JD06 | 22,1347 | 1242,9 | 5,2 | 993,8 | 8,4 | 1,63E-13 | 5,03E-15 | 3,33E-15 | 8,81E-16 | 6,84E-13 | 2,82E-14 | 2,13E-15 | 1,85E-15 | 122515 | 4011 | 855669 | 35595 |
| JD07 | 27,1596 | 1341,2 | 17,8 | 993,8 | 8,4 | 2,19E-13 | 6,14E-15 | 3,33E-15 | 8,81E-16 | 7,66E-13 | 3,32E-14 | 2,13E-15 | 1,85E-15 | 133646 | 3968 | 842984 | 38291 |
| JD08 | 27,8693 | 966,7 | 9,2 | 1043,0 | 16,9 | 2,07E-13 | 6,66E-15 | 3,57E-15 | 8,97E-16 | 1,01E-12 | 3,20E-14 | 4,19E-15 | 1,92E-15 | 122327 | 4141 | 780034 | 25938 |
| JD09 | 26,7302 | 1114,7 | 11,8 | 1043,0 | 16,9 | 2,09E-13 | 6,80E-15 | 3,57E-15 | 8,97E-16 | 8,83E-13 | 3,05E-14 | 4,19E-15 | 1,92E-15 | 128654 | 4394 | 818510 | 29756 |
| JD10 | 27,3794 | 1408,1 | 10,7 | 1043,7 | 9,9 | 2,74E-13 | 7,31E-15 | 3,92E-15 | 9,24E-16 | 9,62E-13 | 2,97E-14 | 1,88E-15 | 1,42E-15 | 165451 | 4660 | 1102473 | 35135 |
| JD11 | 29,7222 | 1334,5 | 12,8 | 1043,7 | 9,9 | 2,44E-13 | 7,13E-15 | 3,92E-15 | 9,24E-16 | 9,68E-13 | 3,93E-14 | 1,88E-15 | 1,42E-15 | 135306 | 4174 | 968156 | 40503 |
| JD12 | 28,5264 | 1283,2 | 19,5 | 990,5 | 3,7 | 2,27E-13 | 6,08E-15 | 4,05E-15 | 9,85E-16 | 7,97E-13 | 3,51E-14 | 2,44E-15 | 2,03E-15 | 131102 | 3741 | 798074 | 37306 |
| JD13 | 27,5505 | 1060,9 | 2,8 | 1043,7 | 9,9 | 2,87E-13 | 8,32E-15 | 3,92E-15 | 9,24E-16 | 1,35E-12 | 3,42E-14 | 1,88E-15 | 1,42E-15 | 172452 | 5248 | 1156531 | 29594 |
| JD14 | 24,0760 | 1019,8 | 8,7 | 1043,7 | 9,9 | 2,09E-13 | 6,20E-15 | 3,92E-15 | 9,24E-16 | 1,03E-12 | 3,10E-14 | 1,88E-15 | 1,42E-15 | 140416 | 4412 | 976245 | 30552 |
| Phy01 | 20,8828 | 1454,6 | 10,6 | 1000,4 | 7,5 | 1,98E-13 | 7,24E-15 | 2,96E-16 | 8,08E-16 | 5,83E-13 | 2,44E-14 | 1,25E-15 | 1,15E-15 | 144189 | 5336 | 905648 | 38446 |
| Phy02 | 21,0793 | 1342,9 | 7,5 | 1000,4 | 7,5 | 1,45E-13 | 5,94E-15 | 2,96E-16 | 8,08E-16 | 5,01E-13 | 2,48E-14 | 1,25E-15 | 1,15E-15 | 104848 | 4375 | 711101 | 35467 |
| SF01 | 10,5144 | 1354,2 | 1,2 | 990,5 | 3,7 | 1,08E-13 | 4,34E-15 | 4,05E-15 | 9,85E-16 | 4,01E-13 | 2,00E-14 | 2,44E-15 | 2,03E-15 | 166057 | 7230 | 1148081 | 57558 |
| SF06 | 13,5190 | -- | -- | -- | -- | 1,88E-13 | 5,72E-15 | 2,90E-15 | 1,30E-15 | -- | -- | -- | -- | 188739 | 7107 | -- | -- |
| SF07 | 17,6760 | -- | -- | -- | -- | 4,83E-13 | 1,15E-14 | 2,90E-15 | 1,30E-15 | -- | -- | -- | -- | 375570 | 11809 | -- | -- |
| SF08 | 16,6830 | -- | -- | -- | -- | 4,93E-13 | 1,29E-14 | 2,90E-15 | 1,30E-15 | -- | -- | -- | -- | 408921 | 13608 | -- | -- |
| SF09 | 13,8380 | -- | -- | -- | -- | 4,59E-13 | 1,41E-14 | 2,90E-15 | 1,30E-15 | -- | -- | -- | -- | 458082 | 16972 | -- | -- |
| SF13 | 18,5330 | -- | -- | -- | -- | 4,60E-13 | 1,38E-14 | 2,90E-15 | 1,30E-15 | -- | -- | -- | -- | 343454 | 12483 | -- | -- |
| SF15 | 18,4200 | -- | -- | -- | -- | 3,00E-13 | 1,35E-14 | 2,90E-15 | 1,30E-15 | -- | -- | -- | -- | 224902 | 11195 | -- | -- |
| SF17 | 20,1420 | -- | -- | -- | -- | 3,10E-13 | 7,90E-15 | 2,90E-15 | 1,30E-15 | -- | -- | -- | -- | 212139 | 6998 | -- | -- |
| SF18 | 18,3590 | -- | -- | -- | -- | 3,87E-13 | 1,01E-14 | 2,90E-15 | 1,30E-15 | -- | -- | -- | -- | 291233 | 9699 | -- | -- |
| SF19 | 25,7009 | 1326,0 | 11,0 | *990,5* | *3,7* | 2,33E-13 | 6,42E-15 | 4,05E-15 | 9,85E-16 | 8,22E-13 | 2,95E-14 | 2,44E-15 | 2,03E-15 | 150052 | 4383 | 944328 | 34945 |
| SF21 | 17,7920 | 1041,1 | 17,2 | *1029,6* | *20,6* | 6,70E-13 | 1,44E-14 | 2,90E-15 | 1,30E-15 | 2,64E-12 | 6,45E-14 | 4,93E-15 | 3,49E-15 | 522310 | 15443 | 3444289 | 101821 |
| SF22 | 20,0940 | 1831,0 | 37,4 | *1029,6* | *20,6* | 7,69E-13 | 1,52E-14 | 2,90E-15 | 1,30E-15 | 1,69E-12 | 4,26E-14 | 4,93E-15 | 3,49E-15 | 530398 | 15012 | 3441383 | 111678 |
| SF23 | 19,4530 | 1269,6 | 109,3 | *1029,6* | *20,6* | 7,50E-13 | 1,72E-14 | 2,90E-15 | 1,30E-15 | 2,34E-12 | 5,73E-14 | 4,93E-15 | 3,49E-15 | 533154 | 16325 | 3403437 | 305233 |
| SF24 | 22,9980 | 976,5 | 19,1 | *1029,6* | *20,6* | 1,13E-12 | 2,69E-14 | 2,90E-15 | 1,30E-15 | 4,70E-12 | 1,14E-13 | 4,93E-15 | 3,49E-15 | 679195 | 21189 | 4447772 | 139160 |
| SF25 | 21,5580 | -- | -- | -- | -- | 2,66E-13 | 6,56E-15 | 2,90E-15 | 1,30E-15 | -- | -- | -- | -- | 169905 | 5519 | -- | -- |
| SF26 | 8,2340 | -- | -- | -- | -- | 7,81E-14 | 3,93E-15 | 1,15E-14 | 2,30E-15 | -- | -- | -- | -- | 112161 | 8144 | -- | -- |
| SF27 | 21,7880 | 1177,6 | 58,6 | *1029,6* | *20,6* | 6,77E-13 | 1,03E-14 | 1,15E-14 | 2,30E-15 | 2,26E-12 | 5,78E-14 | 1,48E-14 | 5,25E-15 | 424449 | 10950 | 2712352 | 152728 |

| Sample ID | Quartz Mass (g) | Total Al (ug)* | Unc. | Laboratory blank Total Al (ug)* | Unc. | 10Be/9Be Ratio | Unc. | Laboratory blank 10Be/9Be Ratio | Unc. | 26Al/27Al Ratio | Unc. | Laboratory blank 26Al/27Al Ratio | Unc. | 10Be Conc. (atoms g^-1) | Unc. | 26Al Conc. (atoms g^-1) | Unc. |
|---|---|---|---|---|---|---|---|---|---|---|---|---|---|---|---|---|---|
| SF28 | 24,3030 | 1378,8 | 46,1 | *1029,6* | *20,6* | 6,44E-13 | 1,08E-14 | 1,15E-14 | 2,30E-15 | 1,89E-12 | 5,05E-14 | 1,48E-14 | 5,25E-15 | 363439 | 9748 | 2379933 | 102528 |
| SF29 | 23,2550 | 1322,4 | 40,5 | *1029,6* | *20,6* | 6,87E-13 | 1,12E-14 | 1,15E-14 | 2,30E-15 | 2,01E-12 | 4,94E-14 | 1,48E-14 | 5,25E-15 | 405197 | 10738 | 2539247 | 100431 |
| SF31 | 22,9260 | -- | -- | -- | -- | 1,01E-12 | 1,90E-14 | 1,15E-14 | 2,30E-15 | -- | -- | -- | -- | 606692 | 16934 | -- | -- |
| SF32 | 23,1550 | -- | -- | -- | -- | 5,69E-13 | 1,05E-14 | 1,15E-14 | 2,30E-15 | -- | -- | -- | -- | 336354 | 9468 | -- | -- |
| SF33 | 21,9680 | 1224,8 | 56,0 | *1029,6* | *20,6* | 7,24E-13 | 1,59E-14 | 1,15E-14 | 2,30E-15 | 2,50E-12 | 6,15E-14 | 1,49E-14 | 5,25E-15 | 452130 | 13728 | 3090841 | 161486 |
| SF34 | 24,8730 | -- | -- | -- | -- | 8,17E-13 | 1,65E-14 | 1,15E-14 | 2,30E-15 | -- | -- | -- | -- | 452101 | 13097 | -- | -- |
| SF35 | 18,8680 | -- | -- | -- | -- | 2,67E-13 | 1,05E-14 | 1,15E-14 | 2,30E-15 | -- | -- | -- | -- | 188637 | 8885 | -- | -- |
| SF36 | 21,8030 | -- | -- | -- | -- | 1,91E-13 | 5,72E-15 | 1,15E-14 | 2,30E-15 | -- | -- | -- | -- | 114698 | 4642 | -- | -- |
| SF37 | 20,7800 | -- | -- | -- | -- | 1,73E-13 | 5,25E-15 | 1,15E-14 | 2,30E-15 | -- | -- | -- | -- | 108390 | 4500 | -- | -- |
| SF38 | 19,3360 | -- | -- | -- | -- | 1,56E-13 | 5,64E-15 | 1,15E-14 | 2,30E-15 | -- | -- | -- | -- | 104080 | 4940 | -- | -- |
| SF39 | 21,9200 | -- | -- | -- | -- | 1,71E-13 | 5,44E-15 | 1,15E-14 | 2,30E-15 | -- | -- | -- | -- | 101253 | 4343 | -- | -- |
| SF40 | 20,4650 | -- | -- | -- | -- | 1,45E-13 | 4,88E-15 | 1,15E-14 | 2,30E-15 | -- | -- | -- | -- | 90170 | 4160 | -- | -- |
| SF41 | 21,4050 | -- | -- | -- | -- | 1,82E-13 | 5,36E-15 | 1,15E-14 | 2,30E-15 | -- | -- | -- | -- | 111377 | 4485 | -- | -- |
| SF43 | 25,7260 | 1111,3 | 7,0 | 1043,0 | 16,9 | 1,82E-13 | 5,97E-15 | 3,57E-15 | 8,97E-16 | 8,81E-13 | 3,09E-14 | 4,19E-15 | 1,92E-15 | 116750 | 4032 | 845972 | 30344 |
| SF44 | 31,3614 | 1168,4 | 13,9 | 1043,0 | 16,9 | 2,27E-13 | 6,56E-15 | 3,57E-15 | 8,97E-16 | 1,03E-12 | 3,60E-14 | 4,19E-15 | 1,92E-15 | 119541 | 3641 | 852110 | 31670 |
| SF45 | 29,2153 | 1206,9 | 8,4 | 1043,7 | 9,9 | 2,31E-13 | 7,02E-15 | 3,92E-15 | 9,24E-16 | 9,15E-13 | 2,74E-14 | 1,88E-15 | 1,42E-15 | 129644 | 4153 | 842538 | 26009 |
| SF46 | 31,2184 | 1535,9 | 14,8 | 1043,7 | 9,9 | 2,46E-13 | 6,42E-15 | 3,92E-15 | 9,24E-16 | 8,02E-13 | 3,38E-14 | 1,88E-15 | 1,42E-15 | 130075 | 3605 | 878995 | 38087 |
| SF47 | 28,3101 | 1131,9 | 5,1 | 1043,0 | 16,9 | 1,95E-13 | 6,13E-15 | 3,57E-15 | 8,97E-16 | 9,29E-13 | 3,02E-14 | 4,19E-15 | 1,92E-15 | 113072 | 3758 | 825792 | 27252 |
| SF48 | 28,6146 | 1217,9 | 5,8 | 1043,0 | 16,9 | 4,29E-13 | 9,72E-15 | 3,57E-15 | 8,97E-16 | 1,78E-12 | 4,30E-14 | 4,19E-15 | 1,92E-15 | 248525 | 5974 | 1682855 | 41677 |
| SF49 | 26,5336 | 1101,7 | 5,9 | 1043,0 | 16,9 | 2,72E-13 | 7,19E-15 | 3,57E-15 | 8,97E-16 | 1,22E-12 | 3,47E-14 | 4,19E-15 | 1,92E-15 | 168774 | 4724 | 1122487 | 32764 |
| SF50 | 28,9184 | 1165,6 | 8,9 | 1043,0 | 16,9 | 3,40E-13 | 7,62E-15 | 3,57E-15 | 8,97E-16 | 1,38E-12 | 3,66E-14 | 4,19E-15 | 1,92E-15 | 194305 | 4640 | 1240443 | 34340 |
| SF51 | 27,6848 | 1001,5 | 14,9 | 1043,0 | 16,9 | 3,49E-13 | 8,11E-15 | 3,57E-15 | 8,97E-16 | 1,63E-12 | 4,28E-14 | 4,19E-15 | 1,92E-15 | 210125 | 5180 | 1313445 | 39716 |
| SF52 | 27,6954 | 1116,4 | 5,7 | 1043,7 | 9,9 | 3,27E-13 | 7,93E-15 | 3,92E-15 | 9,24E-16 | 1,59E-12 | 5,22E-14 | 1,88E-15 | 1,42E-15 | 195281 | 5033 | 1425990 | 47542 |
| SF53 | 29,5795 | 1409,4 | 8,9 | 993,8 | 8,4 | 1,81E-13 | 6,00E-15 | 3,33E-15 | 8,81E-16 | 6,08E-13 | 2,56E-14 | 2,13E-15 | 1,85E-15 | 104061 | 3620 | 644551 | 27521 |
| SF54 | 29,0167 | 1030,1 | 0,8 | 1043,7 | 9,9 | 1,72E-13 | 5,82E-15 | 3,92E-15 | 9,24E-16 | 9,05E-13 | 2,84E-14 | 1,88E-15 | 1,42E-15 | 97300 | 3480 | 715198 | 22522 |
| SF55 | 27,0836 | 1404,3 | 9,7 | 993,8 | 8,4 | 1,61E-13 | 5,81E-15 | 3,33E-15 | 8,81E-16 | 5,83E-13 | 2,67E-14 | 2,13E-15 | 1,85E-15 | 98007 | 3717 | 672362 | 31231 |
| SF56 | 28,2962 | 1467,5 | 6,7 | 993,8 | 8,4 | 1,62E-13 | 5,74E-15 | 3,33E-15 | 8,81E-16 | 5,12E-13 | 2,36E-14 | 2,13E-15 | 1,85E-15 | 94473 | 3517 | 591074 | 27541 |
| SF57 | 24,5233 | 1374,8 | 12,5 | 993,8 | 8,4 | 1,49E-13 | 5,84E-15 | 3,33E-15 | 8,81E-16 | 4,79E-13 | 2,24E-14 | 2,13E-15 | 1,85E-15 | 99578 | 4114 | 597387 | 28615 |

* Total Al in the sample including both native Al in quartz and Al added via carrier and quantified by ICP-OES directly following digestion. For processing blanks prepared at SUERC *(italicised),* total Al are determined from Al spike (not ICP-measured).

All uncertainties are one standard deviation

**Supplementary table 3.**

**Inverse-modelled erosion rates (m Myr$^{-1}$)**

| Sample ID | Median | 1st Quartile | 3rd Quartile |
|---|---|---|---|
| HD01 | 8,2 | 4,3 | 12,4 |
| HD02 | 16,9 | 9,7 | 24,2 |
| HD04 | 6,9 | 3,3 | 10,1 |
| HD06[b] | 5,6 | 2,9 | 6,6 |
| HD08[b] | 4,0 | 2,1 | 5,1 |
| HD09[b] | 4,3 | 2,3 | 4,9 |
| HD10 | 92,9 | 33,6 | 258 |
| HD12 | 69,6 | 29,9 | 207 |
| HD16 | 6,3 | 3,2 | 10,5 |
| HD17 | 10,5 | 5,8 | 15,6 |
| HD28 | 6,0 | 2,9 | 9,9 |
| JD04 | 9,3 | 4,7 | 13,0 |
| JD05 | 2,4 | 1,2 | 5,1 |
| JD06 | 16,4 | 9,7 | 22,2 |
| JD07 | 12,7 | 7,9 | 18,8 |
| JD08 | 22,3 | 13,4 | 33,6 |
| JD09 | 15,4 | 9,5 | 20,2 |
| JD10 | 11,7 | 6,3 | 15,3 |
| JD11 | 18,9 | 11,6 | 25,2 |
| JD13 | 11,1 | 5,4 | 14,3 |
| JD14 | 25,8 | 16,2 | 50,2 |
| Phy01 | 39,4 | 20,6 | 138 |
| Phy02 | 47,8 | 23,8 | 191 |
| SF07[a] | 32,3 | 17,1 | 123 |
| SF08[a] | 17,1 | 9,7 | 26,2 |
| SF09[a] | 9,5 | 5,1 | 14,0 |
| SF13[a] | 23,0 | 12,7 | 51,0 |
| SF15[a] | 39,2 | 16,7 | 94,5 |
| SF17[a] | 39,2 | 16,9 | 87,4 |
| SF18[a] | 61,8 | 26,3 | 192 |
| SF21[b] | 6,1 | 3,3 | 7,3 |
| SF22[b] | 5,8 | 3,0 | 7,2 |
| SF23[b] | 5,6 | 2,9 | 6,9 |
| SF24[b] | 5,2 | 3,1 | 6,4 |
| SF27 | 5,9 | 2,7 | 8,1 |
| SF28 | 6,5 | 2,9 | 9,6 |
| SF29 | 4,9 | 2,2 | 8,4 |
| SF32[a] | 13,6 | 8,0 | 19,5 |
| SF33 | 5,6 | 3,3 | 6,8 |
| SF34[a] | 8,0 | 4,0 | 12,0 |
| SF35[a] | 39,6 | 16,4 | 84,4 |
| SF36[a] | 42,6 | 18,1 | 82,0 |
| SF37[a] | 42,7 | 17,6 | 77,9 |
| SF38[a] | 43,8 | 18,3 | 77,0 |
| SF41[a] | 43,3 | 17,9 | 83,7 |
| SF43 | 24,8 | 14,4 | 41,6 |
| SF44 | 22,2 | 13,5 | 31,8 |
| SF45 | 18,2 | 11,1 | 25,2 |
| SF46 | 17,4 | 10,3 | 23,6 |
| SF47 | 33,4 | 20,0 | 94,1 |
| SF48 | 7,5 | 3,8 | 9,1 |
| SF49 | 9,8 | 5,2 | 14,2 |
| SF50 | 7,6 | 3,9 | 11,5 |
| SF51 | 6,9 | 3,3 | 10,5 |
| SF52 | 10,4 | 5,3 | 12,5 |
| SF53 | 21,9 | 13,0 | 31,7 |
| SF54 | 32,5 | 20,1 | 79,0 |
| SF55 | 19,9 | 11,9 | 27,7 |
| SF56 | 32,6 | 18,6 | 80,3 |
| SF57 | 19,7 | 10,8 | 29,4 |

Simulations using spallation and muon production rates from the online calculators formerly known as the CRONUS-Earth online calculators v.2.3 (See Methods). Attenuation length for spallation is 160 g cm$^{-2}$ and the density is 2650 kg m$^{-3}$. The muonic production rates are scaled to the site elevation using energy-dependent attenuation lengths (Sup. Ref. 5). Production rates of negative vs. fast muons are 0.53/0.47 of the total muon production for 10Be and 0.54/0.46 for 26Al respectively.

Relative uncertainties on concentrations is 0.05 for $^{10}$Be and 0.1 for $^{26}$Al.

[a]Inversion based solely on $^{10}$Be data

[b]Samples from blockfield-covered plateau summit

**Supplementary References**


1. Brook, E. J., Nesje, A., Lehman, S. J., Raisbeck, G. M., & Yiou, F. Cosmogenic nuclide exposure ages along a vertical transect in western Norway: implications for the height of the Fennoscandian ice sheet. *Geology* **24,** 207-210 (1996).
2. Linge, H. et al. In situ $^{10}$Be exposure ages from southeastern Norway: implications for the geometry of the Weichselian Scandinavian ice sheet. *Quaternary Science Review* **25,** 1097-1109 (2006).
3. Goehring, B. M., Brook, E. J., Linge, H., Raisbeck, G. M., & Yiou, F. Beryllium-10 exposure ages of erratic boulders in southern Norway and implications for the history of the Fennoscandian Ice Sheet. *Quaternary Science Review* **27,** 320-336 (2008).
4. Borchers, B. et al. Geological calibration of spallation production rates in the CRONUS-Earth project. *Quaternary Geochronology* **31,** 188-198 (2016).
5. Balco, G., Stone, J. O., Lifton, N. A., & Dunai, T. J. A complete and easily accessible means of calculating surface exposure ages or erosion rates from $^{10}$Be and $^{26}$Al measurements. *Quaternary Geochronology* **3,** 174-195 (2008).
